# Supplementary material for: Vitamin D3 Inhibits the Viability of Breast Cancer Cells In Vitro and Ehrlich Ascites Carcinomas in Mice by Promoting Apoptosis and Cell Cycle Arrest and by Impeding Tumor Angiogenesis
Source: Cancers (Basel). 2023 Oct 2;15(19):4833. doi: 10.3390/cancers15194833 (PMC10571758; doi:10.3390/cancers15194833)
Supplement: Supplementary file 1 [file cancers-15-04833-s001.zip › cancers-2604600-supplementary.pdf]

cancers

MDPI

Vitamin D3 Inhibits the Viability of Breast Cancer Cells In Vitro and Ehrlich Ascites Carcinomas in Mice by Promoting Apoptosis and Cell Cycle Arrest and by Impeding Tumor Angiogenesis

Prashanth Kumar M. Veeresh, Chaithanya G. Basavaraju, Siva Dallavalasa, Preethi G. Anantharaju, Suma M. Natraj, Olga A. Sukocheva and SubbaRao V. Madhunapantula

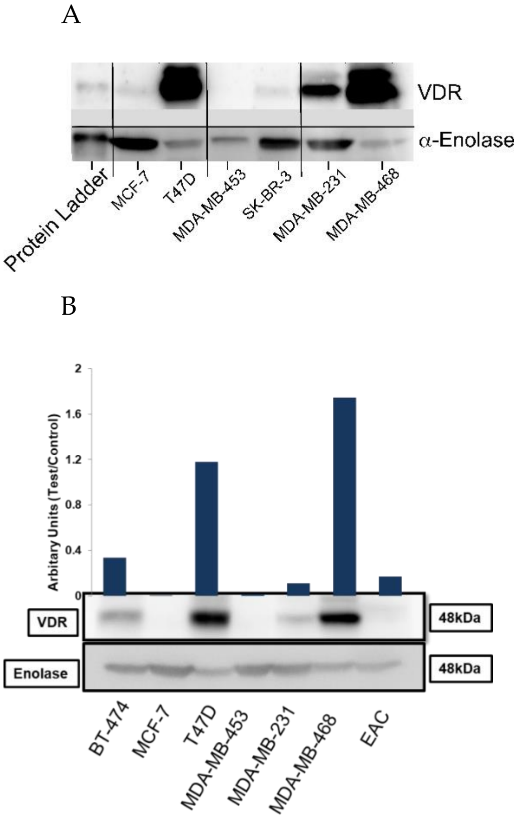

Figure S1. Expression of VDR in breast cancer cell lines harboring or not harboring HER2. Breast cancer cell lines expressing HER2 (BT-474, MDA-MB-453) or not expressing HER2 (MCF-7, T47D, MDA-MB-231 and MDA-MB-468) were grown to confluence and protein lysates harvested using RIPA buffer. About 50micrograms protein/well was loaded and electrophoresed on 10% SDS polyacrylamide gel. The proteins were transferred to PVDF membrane and the expression of VDR and the loading control alpha enolase was measured by probing with respective primary antibodies. Analysis of the data showed elevated expression predominantly in HER2 negative cell lines. Contrary to this general trend the HER2 positive cell line BT-474 and HER2 negative cell line MCF-7 expressed or not expressed the VDR.

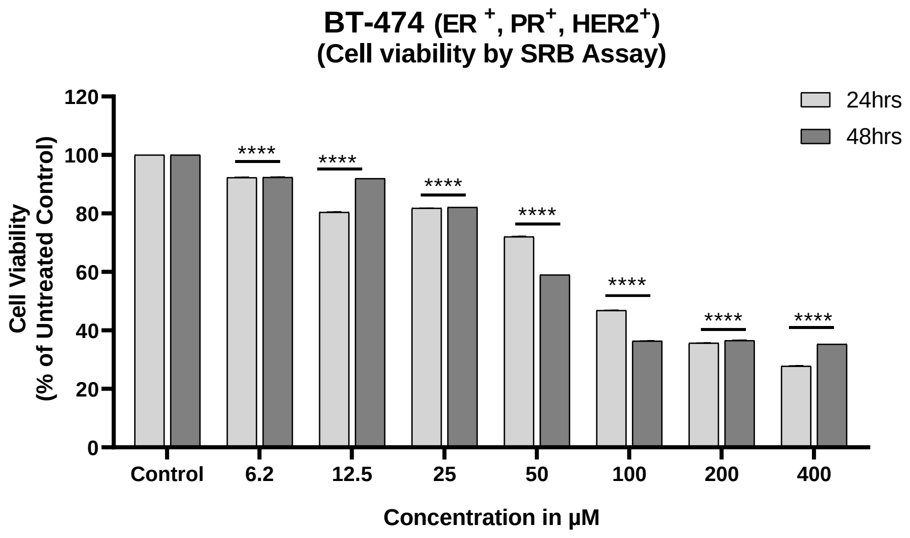

Figure S2. Vitamin-D3 effectively inhibited the viability of triple positive breast cancer cell line BT-474 in vitro. In order to test whether vitamin-D3 could inhibit the viability of even the triple positive cell lines, 1 X 10^4 BT-474 cells / 100 microliters media were plated in a 96-well plate and allowed to grow for about 30h. The cells were exposed to increasing concentration of vitamin-D3 for 24h and 48h, and viability of cells determined using sulforhodamine-B assay. Analysis of the data showed a dose dependent decrease in the viability of BT-474 cells with increasing vitamin-D3 concentration. This indicates that vitamin-D3 inhibits the viability of breast cancer cells irrespective of their HER2/VDR expression. .
